# 

**Published:** 1988

**Authors:** 


Copyright © Clinical Press Ltd 1988.

Published quarterly and distributed free to all doctors i
the West of England. Overseas subscription £12.

### Contents

2 **Foreword**

Martin Mott

3 **Current Prognosis for Childhood Cancer in the Sout West Region**

Martin Mott

5 **Causes of death in a Paediatric Oncology Unit**

David Shortland

1 **Growth and Growth Hormone Secretion followin Cranial and Craniospinal irradiation in children with malignant disease**

Paul Ward

14 **Medulloblastoma in the South West**

Nick Foreman

18 **Management of Pulmonary metastases in Osteo genic Sarcoma**

James Wisheart

22 **Staff hazards in handling Chemotherapy**

Sawson Ritha

26 **Ifosamide**

Barry Pizer

29 **Epirubicin**

Jackie Cornish

31 **Carboplatinum**

Joanna Chambers

34 **Molecular biology of Wilm's Tumour**

Andrew Shaw

38 **Cellular biology of Teratoma**

Keith Brown

41 **Control of Bacteraemia from indwelling centra venous catheters**

Nigel Weightman

42 **Stress and coping in Paediatric Oncology**

Geoffrey Moss

45 **Caring for the dying child and family**

Sally Curnick

47 **Some educational implications of childhood cance**

Chris Colburn

51 **The importance of commmunication with the prim ary care team**

John James

53 **Prediction of Psychosocial Sequelae in families o leukaemic children**

Jan Culling

56 **Children's understanding of their illness**

Carol Kendrick

